# Cucurbitacins from the Leaves of *Citrullus colocynthis* (L.) Schrad

**DOI:** 10.3390/molecules201018001

**Published:** 2015-09-30

**Authors:** Rachid Chawech, Raoudha Jarraya, Cynthia Girardi, Marieke Vansteelandt, Guillaume Marti, Imen Nasri, Claire Racaud-Sultan, Nicolas Fabre

**Affiliations:** 1 Université de Toulouse, UPS, Pharma-DEV, UMR 152, Université Toulouse 3, Faculté des Sciences Pharmaceutiques, F-31062 Toulouse cedex 09, France; E-Mails: chawech.rachid@gmail.com (R.C.); cynthia.girardi@gmail.com (C.G.); marieke.vansteelandt@univ-tlse3.fr (M.V.); guillaume.marti@univ-tlse3.fr (G.M.); 2 Institut de Recherche pour le Développement (IRD), UMR 152 Pharma-DEV, F-31062 Toulouse cedex 09, France; 3 Laboratoire de Chimie des Substances Naturelles, Faculté des Sciences de Sfax, Université de Sfax Route de l’aéroport, BP 1171, 3000 Sfax, Tunisie; E-Mails: raoudhajarraya@yahoo.fr (R.J.); nasriimene@gmail.com (I.N.); 4 INSERM U1043, CNRS U5282, Université de Toulouse, UPS, Centre de Physiopathologie de Toulouse Purpan, F-31300 Toulouse, France; E-Mail: claire.racaud@inserm.fr

**Keywords:** *Citrullus colocynthis*, cucurbitacins, cytotoxicity

## Abstract

Two new tetracyclic cucurbitane-type triterpene glycosides were isolated from an ethyl acetate extract of *Citrullus colocynthis* leaves together with four known cucurbitacins. Their structures were established on the basis of their spectroscopic data (mainly NMR and mass spectrometry). Evaluation of the *in vitro* cytotoxic activity of the isolated compounds against two human colon cancer cell lines (HT29 and Caco-2) and one normal rat intestine epithelial cell line (IEC6), revealed that one of the isolated compounds presented interesting specific cytotoxic activity towards colorectal cell lines.

## 1. Introduction

The plants used in Tunisian traditional medicine are a rich source of pharmacologically active natural products [1,2,3,4,5]. *Citrullus colocynthis* (L.) Schrad. (Cucurbitaceae) is a valuable plant widely distributed in the desert areas of the world including Tunisia [6,7]. This medicinal plant has been used in African and Asian traditional medicines to treat arthritis, diabetes, inflammatory disorders and stomachache [7,8,9,10]. Because of its prominent volume and mass (around 500 g), the fruit (bitter apple) has been studied intensively for its wide range of biological activities including antioxidant, cytotoxic, antidiabetic, antilipidemic, insecticide, antimicrobial and anti-inflammatory properties (for a review see [11]). However, little is known concerning the other parts of the plant and only few articles report antioxidant and antibacterial activities of *C. colocynthis* leaf extracts [10,12,13,14]. The phytochemical content of fruits is well known for bioactive compounds such as cucurbitacins; phenolic acids and flavonoids; pyridine and quinolone-type alkaloids, fatty acids; and a volatile fraction containing small alcoholic and ketonic alkyl chains [11,15,16,17]. Among these derivatives, cucurbitacins and their glycosides have been the most studied since, to date, 20 of these highly oxygenated triterpenoids were isolated from the fruit [11,18,19,20,21,22,23]. These compounds constitute a group of diverse tetracyclic triterpenoid substances, which are well known for their bitterness and toxicity (for a review, see [24]). They possess a broad range of potent biological activities, deriving largely from their cytotoxic and anti-tumor properties [24,25,26,27,28,29,30,31]. In our effort to valorize Tunisian medicinal plants, we decided to investigate the cucurbitacin content of *C. colocynthis* leaves. Therefore, we report here the isolation and structural identification of two new cucurbitacin glycosides (6′-acetyl-2-*O*-β-d-glucocucurbitacin E (**1**) and 25-*p*-coumaroyl-3′-acetyl-2-*O*-β-d-glucocucurbitacin I (**2**)) along with four known cucurbitacin derivatives (cucurbitacin E (**3**), 2-*O*-glucocucurbitacin E (**4**), cucurbitacin I (**5**) and 2-*O*-glucocucurbitacin I (**6**)). Moreover, given the cytotoxic potential of cucurbitacins on colon cancer cells [28], we evaluated the *in vitro* cytotoxic activity of the compounds isolated against colon cancer cell lines Caco-2 and HT29, which exhibit a different mutation phenotype [32], and the non-transformed IEC6 intestinal cell line [33].

## 2. Results and Discussion

**Figure 1:**
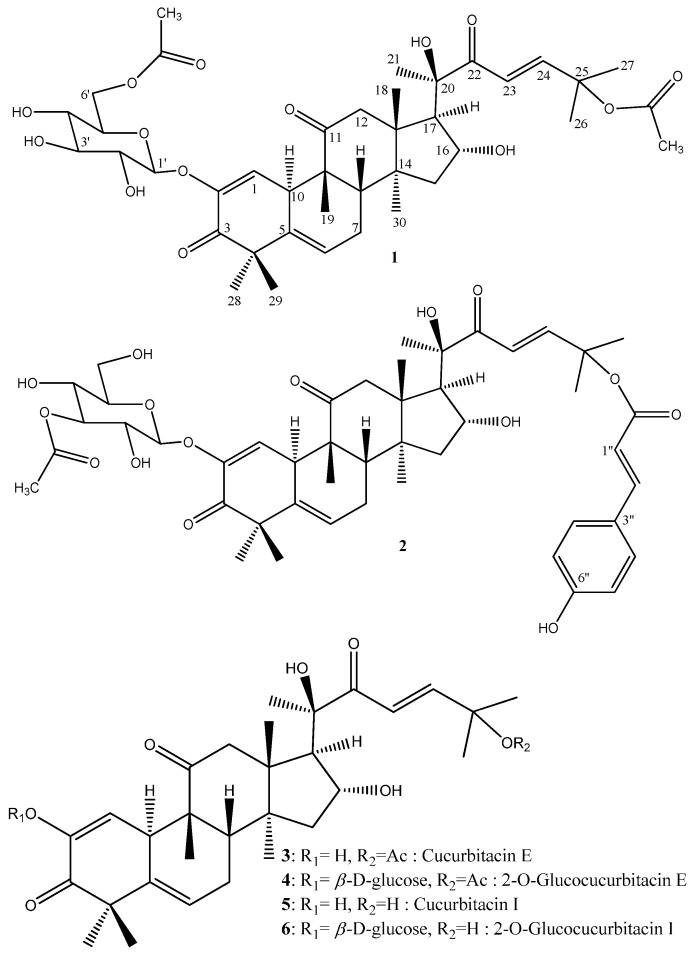
Structures of compounds **1**–**6**.

Cucurbitacins **1**–**6** (Figure 1) were detected in a defatted ethyl acetate leaf extract by TLC interfaced with ESI-MS in positive ionization mode. By comparison with the data in the literature, suspected cucurbitacin candidates were further purified using conventional chromatographic methods.

**Figure 2:**
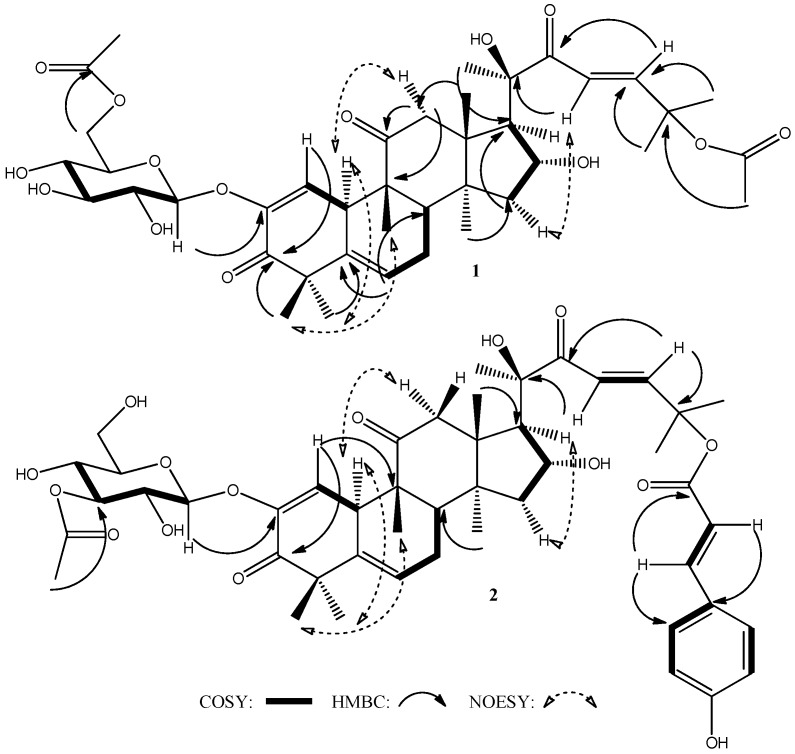
Key COSY (thick bonds), HMBC (solid arrows) and NOESY (dashed arrows) correlations of compounds **1** and **2**.

Compound **1**, a white amorphous powder, had the molecular formula C_40_H_56_O_14_, as established by HR-ESIMS (*m*/*z* 783.3564 [M + Na]^+^; ∆ppm = 0.26) (Figure S1), thus requiring 13 degrees of unsaturation. The UV spectrum (Figure S2) exhibited an absorption band at 229 nm. The IR spectrum of compound **1** (Figure S3) showed absorption bands at 1736, 1691 and 1655 cm^−1^ which have been ascribed to carbonyl ester, ketone, and enone, respectively, and broad bands at 3347 and 1029 cm^−1^ suggestive of a glycoside moiety. The *J*-modulated ^13^C-NMR spectrum of compound **1** revealed the presence of 40 signals (Table 1 and Figure S4) corresponding to 10 methyls, 4 methylenes, 13 methines and 13 quaternary carbons. Among them, four signals are easily attributable to two acetyl groups (δ_C_ 22.0, 170.3 and 21.0, 171.9) and six peaks confirm the presence of a sugar structure (5 CH-OH between 69 and 100 ppm and a CH_2_-O at 65.4 ppm). Thirty other carbons can be assigned to a highly oxygenated triterpene skeleton including three keto groups at δ_C_ 198.2, 202.5 and 213.1 ppm, two quaternary oxygen-bearing carbons at δ_C_ 78.2 and 79.3 and one CH-O at δ_C_ 71.2. Other features such as eight methyl groups, six olefinic carbons and the high-field value of a methylene at δ_C_ 23.6 (C-7) ppm are thus characteristic attributes of a cucurbitane skeleton [34]. The ^1^H-NMR spectrum showed signals (Table 1 and Figure S5) assignable to a β-glucopyranosyl moiety (anomeric proton H-1′ at δ_H_ 4.65 (d, *J* = 7.7 Hz)), eight methyls at δ_H_ 1.01, 1.05, 1.25, 1.32, 1.40, 1.45, 1.56, 1.59 (all s) and one methine bearing an oxygen function (δ_H_ 4.40 (ddd, 3.2, 3.6, 7.2 Hz, βH-16)). Other features include protons attributable to two trisubstituted olefins (δ_H_ 5.80 (m) H-6 and 6.21 (d, 2.0 Hz) H-1)), a *trans*-olefin pair (δ_H_ 6.49, 7.07 (both d, 15.7 Hz), H-23, H24)) together with two acetyl groups (δ_H_ 2.03 and 2.12 both s). At this stage, all NMR signals are in good agreement with those of cucurbitacin I substituted by a glucose moiety and two acetyl groups [23,34,35]. Key and unambiguous HMBC cross-peaks (Figure 2, Figures S6 and S7) observed between the anomeric proton H-1′ and C-2 and the methyl of one acetyl with C-25 led us to identify compound **1** as a cucurbitacin E 2-*O*-β-d-glucopyranoside substituted with an acetyl group. The latter was positioned at C-6′ of the glucose on the basis of the cross-peaks observed between protons H-6′ and the carbonyl of the acetyl. Total assignments of carbons and protons were achieved by HMBC, HSQC, ^1^H-^1^H COSY (Figures S6–S9 in supplementary information, respectively) and by comparison with a standard of cucurbitacin E. The stereostructure of cucurbitane skeleton in compound **1** was characterized by NOESY experiment (Figure 2 and Figure S10) and the comparison of key chemical shifts and coupling constants with literature. Thus, a coupling constant of 7.2 Hz for H-16 and H-17 is representative of β and α positions for H-16 and H-17, respectively [35,36]. The stereostructure of the C-20 position was deduced by comparison of the ^13^C-NMR data (δ_C_ 78.2 for C-20) with authentic glucocucurbitacin E and the literature’s NMR data for glucocucurbitacins I and E recorded in the same solvent (CDCl_3_ [23]) and was deduced to be *R* oriented. Concerning the configurations of the other asymmetrical carbons in the tetracyclic skeleton, careful sifting of literature revealed that naturally occurring cucurbitacins all keep the same configuration at C-8, C-9, C-10, C-13 and C-14. This finding is logical since all these compounds are biogenically derived from the rearrangement of the same triterpene precursor (protosteryl cation [37]). Thus, the structure of compound **1** was assigned as 6′-acetyl-2-*O*-β-d-glucocucurbitacin E.

Compound **2** was obtained as a yellow amorphous powder. The HR-ESIMS revealed a molecular ion peak at *m*/*z* 887.3823 [M + Na]^+^ consistent with the molecular formula C_47_H_60_O_15_ (∆ppm = −0.11) (Figure S11), possessing 18 degrees of unsaturation. The UV spectrum (Figure S12) exhibited absorption bands at 229 and 312 nm suggesting the presence of a conjugated aromatic ring. The IR spectrum of compound **2** (Figure S13) is very similar to that of compound **1**, particularly concerning the presence of absorption bands at 3406 cm^−1^ due to hydroxyls, and at 1705, 1684 and 1633 cm^−1^ that are attributable to carbonyl ester, ketone and enone, respectively, thereby indicating a highly oxygenated compound. Proton and ^13^C-NMR spectra displayed signals—as reported in Table 1 and Figures S14 and S15, respectively—that could be assigned to a glucocucurbitacin substituted by an acetyl group and another ester whose signals are located in the aromatic and olefinic regions. The latter was identified as a *p*-coumaroyl moiety based on its characteristic NMR data and confirmed by a neutral loss of 146.0370 u (C_9_H_6_O_2_) (ion at *m*/*z* 741.3453, Figure S11) in the positive ion HR-ESIMS spectrum of compound **2**. The position of the acetyl was established from the cross-peak observed in the HMBC spectrum (Figure 2, Figures S16 and S17) between the protons of its methyl group and the C-3′ of the glucose moiety, itself linked to the cucurbitane core on C-2 (Figure 2 and Figure S17) as for compound **1**. Owing to the lack of correlation observed in the HMBC spectrum of compound **2** between the *p*-coumaroyl moiety and the cucurbitane skeleton, the location of this esterification was deduced from a thorough review of the ^13^C-NMR data of different cucurbitacins. Thus, a δ_C_ appears between 71 and 73 ppm for C-25 hydroxylated cucurbitacins (such as cucurbitacin I with C-25 at δ_C_ 71.3) and a downfield δ_C_ around 79–80 for the C-25 esterified derivatives [23,35]. Noting a δ_C_ of 79.1 for the C-25 of compound **2** and on the basis of the above-mentioned evidence, the structure of compound **2** was determined to be 25-*p*-coumaroyl-3′-acetyl-2-*O*-β-d-glucocucurbitacin I.

To our knowledge, compounds **1** and **2** are new natural cucurbitacins and this is the first time that a coumaroyl-derived cucurbitacin is described. However, it is worth noting that compound **1** is the acetylated derivative of the well-known glucocucurbitacin E (**4**). In order to verify if these compounds are not artifacts due to solvent extraction (EtOAc), LC-MS analysis of a crude extract of the leaves of *C. colocynthis* prepared with MeOH was undertaken. Both derivatives **1** and **2** were again easily detected in the MeOH extract thus validating their natural occurrence (Figures S21 and S22 respectively).

**Table 1:** ^1^H- and ^13^C-NMR spectroscopic data for 6′-acetyl-2-*O*-β-d-glucocucurbitacin E (**1**) (500 MHz, CDCl_3_) and 25-*p*-coumaroyl-3′-acetyl-2-*O*-β-d-glucocucurbitacin I (**2**) (400 MHz, CD_3_OD).

Position	1			2		
	δ_H_ (*J* in Hz)	δ_C_	Type	δ_H_ (*J* in Hz)	δ_C_	Type
1	6.21 d (2.0)	123.8	CH	6.11 d (2.3)	121.5	CH
2		145.6	C		145.9	C
3		198.2	C		198.2	C
4		48.6	C		48.9	C
5		135.8	C		136.8	C
6	5.80 m	121.2	CH	5.83 m	120.4	CH
7a	2.41 dd (11.6, 7.2)	23.6	CH_2_	2.09 m	23.2	CH_2_
7b	2.06 d (11.6)			2.36 m		
8	2.05 m	41.5	CH	2.02 m	41.9	CH
9		48.7	C		48.7	C
10	3.50 brs	35.3	CH	3.67 brs	35.1	CH
11		213.1	C		214.8	C
12a	2.74 d (14.7)	48.9	CH_2_	2.62 d (14.8)	47.9	CH_2_
12b	3.26 d (14.7)			3.32 d (14.8)		
13		50.4	C		48.5	C
14		48.1	C		50.7	C
15a	1.49 d (12.9)	45.5	CH_2_	1.45 m	45.1	CH_2_
15b	1.91 dd (12.9, 3.2)			1.87 m		
16	4.40 ddd (3.2, 3.6, 7.2)	71.2	CH	4.59 m	70.5	CH
17	2.50 d (7.2)	58.2	CH	2.59 d (7.0)	58.7	CH
18	1.01 s	19.9	CH_3_	0.89 s	19.5	CH_3_
19	1.05 s	20.2	CH_3_	0.99 s	19.3	CH_3_
20		78.2	C		78.9	C
21	1.45 s	23.9	CH	1.43 s	24.0	CH_3_
22		202.5	C		203.9	C
23	6.49 d (15.7)	120.5	CH	6.86 d (16.0)	121.3	CH
24	7.07 d (15.7)	152.0	CH	7.01 d (16.0)	150.5	CH
25		79.3	C		79.7	C
26	1.56 s	26.4	CH_3_	1.56 s	25.5	CH_3_
27	1.59 s	25.8	CH_3_	1.58 s	25.1	CH_3_
28	1.32 s	20.3	CH_3_	1.31 s	19.5	CH_3_
29	1.25 s	27.8	CH_3_	1.27 s	26.8	CH_3_
30	1.40 s	18.3	CH_3_	1.40 s	17.4	CH_3_
CH_3_-CO	2.03 s	22.0	CH_3_	2.01 s	20.6	CH_3_
CH_3_-CO		170.3	C		170.6	C
CH_3_-CO	2.12 s	21.0	CH_3_			
CH_3_-CO		171.9	C			
1′	4.65 d (7.7)	100.0	CH	4.70 d (7.2)	99.7	CH
2′	3.55 m	72.6	CH	3.44 dd (7.2)	72,9	CH
3′	3.61 m	74.5	CH	3.46 m	74.6	CH
4′	3.66 m	75.2	CH	3.41 m	69.6	CH
5′	3.50 m	69.2	CH	3.70 m	75.9	CH
6′a	4.20 dd (12.9, 3.8)	65.4	CH_2_	4.36 m	62.9	CH_2_
6′b	4.55 dd (12.8, 4.0)			4.68 m		
C=O					169.3	C
1″				6.50 d (15.8)	114.0	CH
2″				7.64 d (15.8)	145.8	CH
3″					126.4	C
4″, 8″				7.43 d (8.7)	129.8	CH
5″, 7″				6.80 d (8.7)	115.7	CH
6″					161.2	C

The known cucurbitacins (Figure 1) were identified by comparison of their NMR data with the literature as cucurbitacin E (also named α-elaterin) (**3**) [23,34], 2-*O*-β-d-glucocucurbitacin E (colocynthin, elaterinide) (**4**) [22], cucurbitacin I (elatericin B) (**5**) [23,34] and 2-*O*-β-d-glucocucurbitacin I (**6**) [23]. They are all described for the first time in the leaves of *C. colocynthis*.

**Figure 3:**
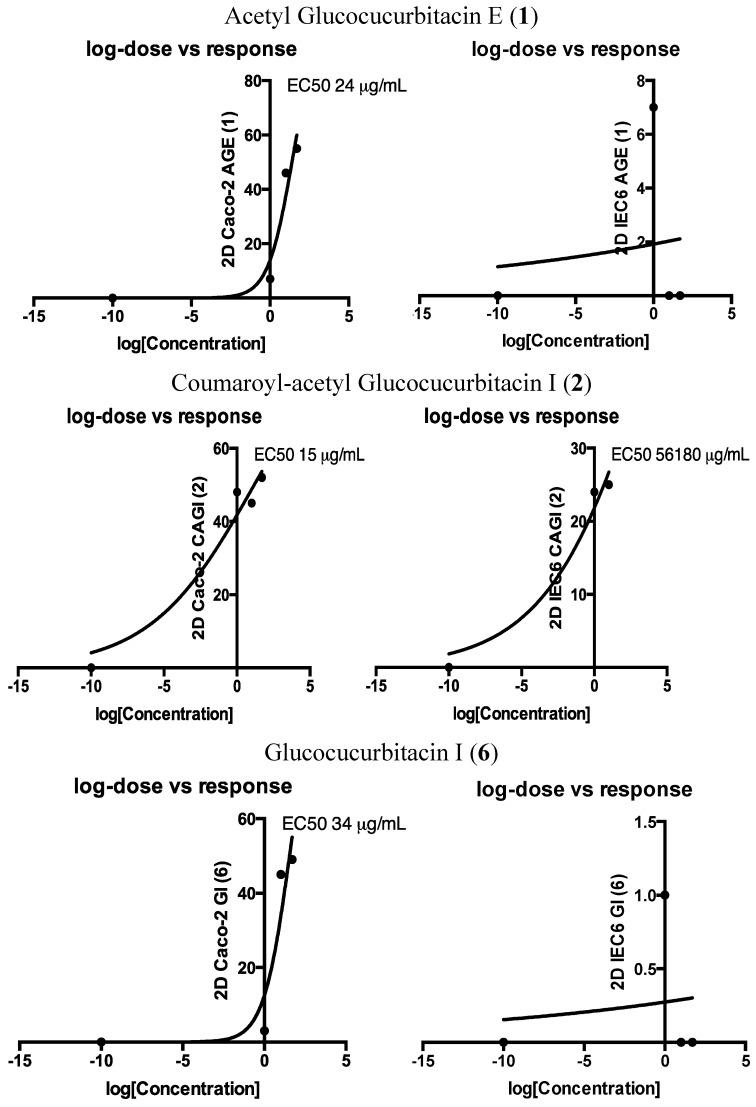
Log-dose response and EC_50_ of compounds **1**, **2** and **6**.

Cytotoxicity of compounds **1**–**6** was evaluated on three cell lines. Tumor cell lines HT29 and Caco-2 (both human colorectal cancer lines) and non-transformed cell line IEC6 (rat small intestine) were grown in 2D or 3D culture conditions 48 h before addition of the compounds to be tested. After 48 h of incubation, cell viability was measured by MTS. As shown in Table 2, in 2D culture conditions, only compounds **1**, **2** and **6** (bold values) induced a significant cytotoxicity (−45%) for Caco-2 cells at low concentrations (1 and 10 µg/mL). At these concentrations, compound **2** was also cytotoxic (−24%) for IEC6 cells. Since 3D culture was reported to be more relevant than 2D culture to study cell survival [38], compounds **1**, **2** and **6** were applied on epithelial spheroids formed by HT29, Caco-2 or IEC6 cells. As shown in Table 2, in 3D culture conditions, only compound **2** was cytotoxic at 1 µg/mL for both HT29 (−32%) and Caco-2 (−19%) spheroids. Importantly, under these conditions, compound **2** was not cytotoxic for IEC6 cells. The dose-response curves (EC_50_) of the active derivatives **1**, **2** and **6** in 2D culture are displayed in Figure 3. This representation clearly demonstrates their specific activity against cancerous cells and the higher cytotoxic potential of compound **2**.

**Table 2:** Cytotoxic activities of compounds **1**–**6**.

µg/mL	HT29 (% of Variation *vs.* Control, Mean ± SD)				Caco-2 (% of Variation *vs.* Control, Mean ± SD)				IEC6 (% of Variation *vs.* Control, Mean ± SD)			
	1	10	50	100	1	10	50	100	1	10	50	100
**2D culture** (3 assays)												
Acetyl Glucocucurbitacin E (**1**)	4.9 ± 0.1	−7.4 ± 0	−4.1 ± 0.1	−0.2 ± 0	−6.7 ± 0.1	−45.5 ± 0.1	−55.4 ± 0	−53.7 ± 0	−6.7 ± 0	0 ± 0	15.5 ± 0	23.2 ± 0
Coumaroyl-acetyl Glucocucurbitacin I (**2**)	−6.4 ± 0.1	−4.1 ± 0	−4.7 ± 0.1	−3.8 ± 0.1	−47.7 ± 0.1	−45.2 ± 0.1	−52.1 ± 0	−35.8 ± 0	−23.7 ± 0	−24.7 ± 0	−2.2 ± 0	7.3 ± 0
Cucurbitacin E (**3**)	8.5 ± 0.2	4.8 ± 0	13.9 ± 0	16.4 ± 0	−11.4 ± 0	7.1 ± 0.1	−8.0 ± 0	−0.3 ± 0.1	−1.2 ± 0	7.7 ± 0	20.9 ± 0	27.9 ± 0
Glucocucurbitacin E (**4**)	11.4 ± 0.1	−0.1 ± 0.1	−9.7 ± 0.2	7.5 ± 0.1	−2.7 ± 0.2	11.1 ± 0.1	4.4 ± 0	−1.1 ± 0	8.1 ± 0	7.2 ± 0	36.0 ± 0	29.3 ± 0
Cucurbitacin I (**5**)	18.8 ± 0.1	6.3 ± 0	5.8 ± 0.1	10.7 ± 0	−11.0 ± 0.1	1.9 ± 0.1	−3.1 ± 0	−1.4 ± 0	−1.8 ± 0	8.7 ± 0.1	2.2 ± 0.1	−14.0 ± 0.1
Glucocucurbitacin I (**6**)	2.7 ± 0.1	2.3 ± 0	−1.6 ± 0.1	2.8 ± 0	−2.6 ± 0.1	−45.3 ± 0.1	−49.1 ± 0	−55.4 ± 0	−0.8 ± 0	4.0 ± 0	10.7 ± 0	16.3 ± 0.1
**3D culture** (2 assays)												
Acetyl Glucocucurbitacin E (**1**)	0 ± 0.1	−24.0 ± 0.2	−6.2 ± 0.4	nd	−3.5 ± 0.4	−3.3 ± 0.1	−23.0 ± 0.1	nd	−13.2 ± 0.1	2.0 ± 0	−17.8 ± 0.3	nd
Coumaroyl-acetyl Glucocucurbitacin I (**2**)	−32.5 ± 0.1	−31.9 ± 0.1	−51.7 ± 0.1	nd	−19.0 ± 0.2	−10.1 ± 0.1	−54.0 ± 0.1	nd	5.7 ± 0.3	2.0 ± 0	−24.4 ± 0.1	nd
Glucocucurbitacin I (**6**)	23.2 ± 0.1	1.2 ± 0.3	1.8 ± 0.4	nd	−1.5 ± 0.2	−1.7 ± 0.4	−12.1 ± 0.2	nd	−2.6 ± 0.1	−5.4 ± 0.1	−9.1 ± 0.1	nd

nd: not determined.

In our experimental conditions, glycosylated cucurbitacins improved cytotoxicity towards colorectal cancer cell lines. Whereas cucurbitacin I (**5**) has been previously shown to have cytotoxic activity against SW480 [31] and Colo205 [39] colorectal cancer cell lines, its glucoside derivative (**6**) was shown here to be specifically and strongly cytotoxic for Caco-2 cells. HT29 cells were not sensitive to glucocucurbitacin I (**6**) nor to other cucurbitacins used in 2D culture. This resistance may be secondary to activated kRas mutant expression in HT29 cells, which can protect colorectal cancer cells from cucurbitacin-induced apoptosis [40]. However, in 3D culture, HT29 cells were specifically decreased after treatment with compound **2** from a concentration of 1 µg/mL. This coumaroyl derivative of glucocucurbitacin I was also cytotoxic for Caco-2 cells at low concentration but not for non-cancerous cells IEC6.

Cucurbitacins can induce growth arrest and apoptosis in colorectal cancer cells [31] through the targeting of the JAK/STAT3 signaling pathway [28]. Moreover, glucocucurbitacins display interesting antioxidant capacities and free radical-scavenging activities, which increase the therapeutic potential of cucurbitacins [41]. It has been shown that the coumaroyl structure can influence the cytotoxic activity of natural compounds [42], and our results suggest that coumaroyl-glycosyl cucurbitacins might have interesting cytotoxic activity to target cancer colorectal cell lines without deleterious effects to normal cells.

## 3. Experimental Section

### 3.1. General Information

Optical rotations were determined at 25 °C on a JASCO (Tokyo, Japan) P2000 polarimeter. UV spectra were recorded on a Thermo Scientific (San Jose, CA, USA) UV-Vis Helios Omega spectrophotometer. IR spectra were taken on a Perkin-Elmer (Waltham, MA, USA) 100 FT-IR spectrometer. NMR spectra were obtained with Bruker (Munich, Germany) instruments (Avance 500 with cryoprobe, 400 and 300 MHz) in CDCl_3_ or CD_3_OD. Chemical shifts are reported as δ values with TMS as the internal standard and the coupling constants (*J*) are in Hz. HR-ESIMS spectra were recorded on Waters (Milford, MA, USA) GTC Premier spectrometer. UHPLC-ESIMS profiles were obtained on a Waters QTOF-MS Xevo G2 instrument hyphenated with a UHPLC Waters. Semi-preparative HPLC purifications were conducted with a Phenomenex column (Luna C18, 5 μm, 10 × 150 mm, 100 Å, injection loop 500 µL), eluted at a flow rate of 3 mL/min. They were carried out using a Hitachi-Merck apparatus (Hitachi, Tokyo, Japan) consisting of a quaternary gradient LC pump LaChrom L-7100, a diode array detector LaChrom L-7455 and a D-7000 interface, all controlled by the D-7000 HSM software. MPLC separations were carried out with Büchi (Flawil, Switzerland) pump and columns (15 × 100 mm). Silica gel (6–35 μm, 60 Å) for MPLC columns was purchased from SDS (Peypin, France). Preparative TLC G-200 UV_254_ (20 × 20 cm, layer 2.0 mm) plates were from Merck (Darmstadt, Germany). Samples were deposited with the help of a Camag (Muffenz, Switzerland) ATS4 model autosampler piloted by WinCATS 1.3.3 software (Camag). TLC-MS was performed using a Camag TLC-MS interface coupled with a mass spectrometer (LCQ DECA XP max, Thermo Finnigan, San Jose, CA, USA) operating in ESI positive ionization mode. Commercial cucurbitacin E was purchased from Sigma-Aldrich (Saint Louis, MO, USA).

### 3.2. Plant Material

The leaves of *Citrullus colocynthis* were collected near Ben Guerdene, in southeastern Tunisia, in May 2012 and authenticated by Prof. Mohamed Chaieb, Department of Biology, University of Sfax. A voucher specimen (LCSN 115) was deposited in the Herbarium of the laboratory of chemistry of natural substances (LCSN), Department of Chemistry, Faculty of Sciences, University of Sfax, Tunisia.

### 3.3. Extraction and Isolation

The air-dried leaves (300 g) were ground and successively macerated thrice with 2 L of *n*-hexane, ethyl acetate and methanol at room temperature for 24 h. The combined extracts were concentrated under reduced pressure on a Büchi Rotavapor^®^ at 40 °C to yield 2.5, 11.0 and 15.0 g of *n*-hexane, EtOAc and MeOH extracts, respectively. The ethyl acetate dry extract was washed with *n*-hexane to remove chlorophylls, leading to a non-polar fraction (4.0 g) and a polar fraction (6.8 g). One gram of the polar fraction was subjected to C18 reversed-phase silica gel column chromatography containing 46 g of silica. The elution was conducted with increasing proportions of H_2_O in CH_3_CN (CH_3_CN:H_2_O 100:0 → 90:10 → 85:15 → 80:20 → 70:30 → 60:40 → 50:50 → 0:100) and yielded nine fractions: F1 (104 mg), F2 (70 mg), F3 (55 mg), F4 (30 mg), F5 (50 mg), F6 (50 mg), F7 (35 mg), F8 (20 mg) and F9 (200 mg). F6 (50 mg) was subjected to MPLC column to obtain 2-*O*-β-d-glucocucurbitacin I (**6**, 10 mg) and sub-fraction 6A (7 mg). F7 (35 mg) was purified by semi-preparative HPLC (H_2_O:CH_3_OH 40:60) at 3 mL/min) to give 2-*O*-β-d-glucocucurbitacin E (**4**, 5 mg, Rt = 9.8 min), cucurbitacin I (**5**, 4 mg, Rt = 11.6 min) and compound **2** (6 mg, Rt = 24.0 min). The chlorophyll-free ethyl acetate extract (2.0 g) was subjected to column chromatography on silica gel (60 Å, 70–200 µm), using binary solvent systems of increasing polarity (from *n*-hexane:EtOAc 100:0 to 0:100 then from EtOAc:MeOH 100:0 to 0:100) to afford 15 fractions. Fraction 5 (130 mg, *n*-hexane:EtOAc 2:8) was washed with dichloromethane to afford cucurbitacin E (**3**, 50 mg). Fraction 10 (80 mg, EtOAc:MeOH 7:3) was purified by preparative TLC plates to yield compounds **1** (4 mg), **2** (7 mg) and **6** (5 mg).

### 3.4. Physical Data of New Compounds

*6′-Acetyl-2*-*O*-β-d-*glucocucurbitacin E* (**1**). White amorphous powder; ${\left[\alpha \right]}_{D}^{25}$−20 (*c* 0.0005, MeOH); UV (MeOH) λ_max_ (nm): 229; IR cm^−1^: 3347, 2926–2854, 1736, 1691, 1655, 1601, 1029; ^1^H-NMR (CDCl_3_, 500 MHz) and ^13^C-NMR (CDCl_3_, 125 MHz) spectral data see Table 1; positive HR-TOF-MS: *m*/*z* 783.3564 [M + Na]^+^ (calcd for C_40_H_56_O_14_Na^+^, 783.3562).

*25-p-Coumaroyl-3′-acetyl-2*-*O*-β-d-*glucocucurbitacin I* (**2**). Yellow amorphous powder; ${\left[\alpha \right]}_{D}^{25}$–36.6 (*c* 0.0005, MeOH), UV (MeOH) λ_max_ (nm): 229, 312; IR cm^−1^: 3406, 2983, 2919, 1705, 1684, 1633, 1615, 1079; ^1^H-NMR (CD_3_OD, 400 MHz) and ^13^C-NMR (CD_3_OD, 100 MHz) spectral data see Table 1; positive HR-TOF-MS: *m*/*z* 887.3823 [M + Na]^+^ (calcd for C_47_H_60_O_15_Na^+^, 887.3824).

### 3.5. Cytotoxicity Assay

HT29 (ATCC-HTB-38) and Caco-2 (ATCC-HTB-37; LGC standards authentication certificate) cells from human colorectal cancer, and IEC6 (ATCC-CRL-1592) cells from rat small intestine, were from ATCC (LGC Standards, Molsheim, France) and were cultured in Dulbecco’s modified Eagle’s medium (DMEM Cat. No. 31966 with Glutamax and 1 mM sodium pyruvate) supplemented with 100 U/mL penicillin/streptomycin and 10% fetal calf serum (FCS) without complement. Caco-2 culture medium was supplemented by 1% non-essential amino acids. All cell culture reagents were from Invitrogen (Carlsbad, CA, USA). The Caco-2 cell line was obtained from a 72-year-old male patient and exhibits APC mutation but wild-type BRAF. The HT29 cell line comes from a 44-year-old female patient and exhibits both APC and BRAF mutations. All cell lines were cultured at 37 °C and 5% CO_2_ until reaching 90% confluence and medium was changed every 2 days. Cells were used for experiments before the 4th passage after thawing (culture duration = 1 month). Forty-eight hours before addition of compounds, cells were plated in a 96-well plate (80,000 cells/well) with DMEM and 10% FCS or embedded in Matrigel for 3D culture. Spheroids of HT29, Caco2 or IEC6 were obtained from 1 × 10^4^ cells embedded in 4 µL Matrigel seeded on top of 20 µL polymerized Matrigel in 48-well plates. After 30 min, DMEM with 10% FCS was added. Spheroids were observed daily using an inverted microscope (Apotome) (Zeiss Axio-observer, HXP120, Carl Zeiss AG, Jena, Germany) to follow their growth. Forty-eight hours after seeding, spheroids showed round structures. Compounds **1**–**6** were dissolved in EtOH 10% to obtain an initial concentration of 10 mg/mL. Then, the compounds were incubated with cells (triplicates and duplicates in 2D and 3D culture, respectively) in DMEM with 5% FCS at increasing concentrations of 1, 10, 50 and 100 µg/mL for 48 h. After, the cells were incubated with MTS-tetrazolium compound (20 µL/well of 96-well plate or 40 µL/well of 48-well plate and Cell Titer 96 Aqueous One Solution assay, Promega, Lyon, France) for 4 h and the colorimetric analysis of surviving cells was made by spectrophotometry (Varioskan Flash, Thermo Fisher Scientific, Illkirch, France). The cytotoxicity of compounds **1**–**6** was then estimated by comparing the variation of cell survival between treated and control assays (EtOH 10%) and was expressed as percentage.

### 3.6. LC-MS Profiles

LC-MS profiles of crude extracts were acquired using a UHPLC-QTOF-MS equipped with an ESI source (QTOF-MS Xevo G2, Waters). Plant extracts were separated using a UPLC BEH C18 Acquity column (150 × 2.1 mm i.d., 1.7 µm) with a gradient from 95% water (+0.1% formic acid) to 95% CH_3_CN (+0.1% formic acid) over 30 min at a flow rate of 460 µL/min. The QTOF-MS was operated both in NI and PI mode at a resolution of approximately 10,000 (full width half maximum). The data were acquired over an *m*/*z* range of 50–1000 in full scan mode. The capillary and cone voltages were set to 2.5 kV and 40 V, respectively. The source temperature was maintained at 120 °C, and the desolvation and cone gas flows were set to 900 L/h at 350 °C and 20 L/h, respectively.

## 4. Conclusions

The chemical investigation of *Citrullus colocynthis* leaf ethyl acetate extract resulted in the isolation of two new cucurbitacins named 6′-acetyl-2-*O*-β-d-glucocucurbitacin E (**1**) and 25-*p*-coumaroyl-3′-acetyl-2-*O*-β-d-glucocucurbitacin I (**2**) along with four known other cucurbitane derivatives (**3**–**6**). The new coumaroyl cucurbitacin derivative **2** displayed interesting specific cytotoxic activity towards colorectal cell lines. Nonetheless, these preliminary results should be confirmed on other normal and cancer cell lines and on *ex vivo* and *in vivo* cancerous tissues, before considering further development for these compounds.

## Supplementary Materials

Supplementary materials can be accessed at: http://www.mdpi.com/1420-3049/20/10/18001/s1.
