# Supplementary material for: Cucurbitacins from the Leaves of Citrullus colocynthis (L.) Schrad
Source: Molecules. 2015 Sep 30;20(10):18001–15. doi: 10.3390/molecules201018001 (PMC6332406; doi:10.3390/molecules201018001)
Supplement: Supplementary file 1 [file molecules-20-18001-s001.pdf]

Supplementary Materials

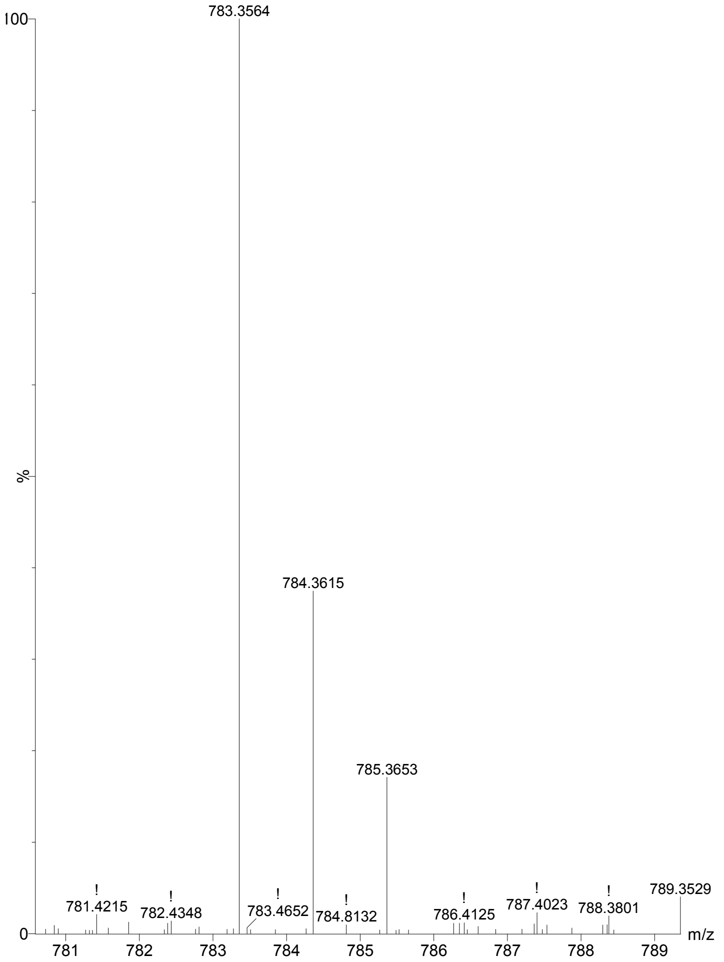

Elemental Composition Report							
Mass	Calc. Mass	mDa	PPM	DBE	i-FIT	i-FIT (Norm)	Formula
783.3564	783.3568	−0.4	−0.5	12.5	73.7	0.0	C40 H56 O14 Na

Figure S1. HR-ESI(+)MS spectrum of 6′-acetyl-2-*O*-β-D-glucocucurbitacin E (**1**) obtained by direct infusion of a methanolic solution (100 μg/mL). Elemental composition report was given by MassLynx V4.1 software.

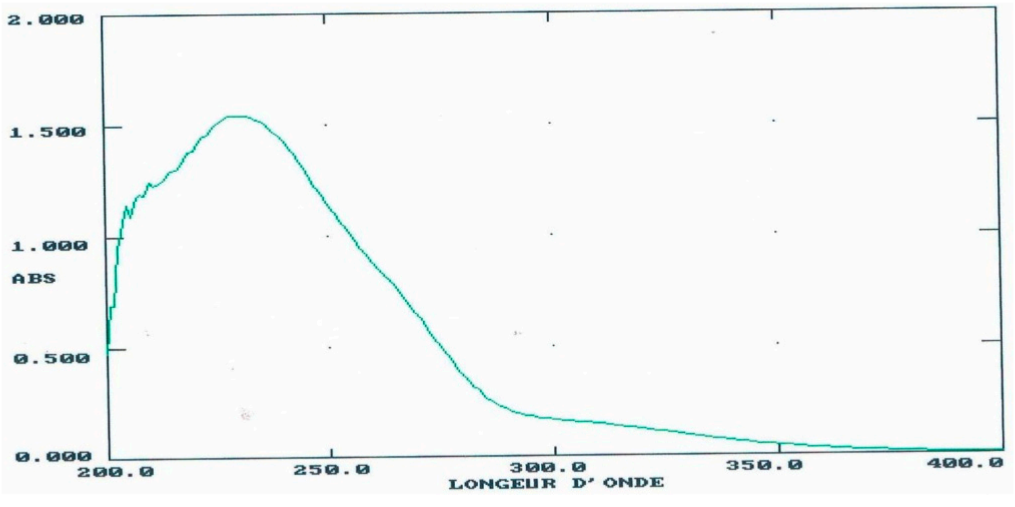

Figure S2. UV spectrum of 6′-acetyl-2-*O*-β-D-glucocucurbitacin E (**1**) in MeOH.

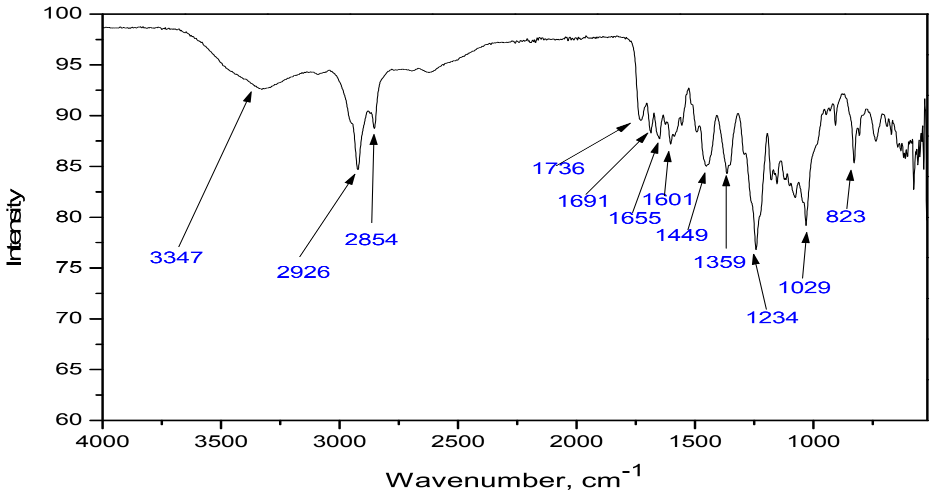

Figure S3. IR spectrum of 6′-acetyl-2-*O*-β-D-glucocucurbitacin E (**1**) recorded on a Perkin-Elmer 100 FT-IR spectrometer.

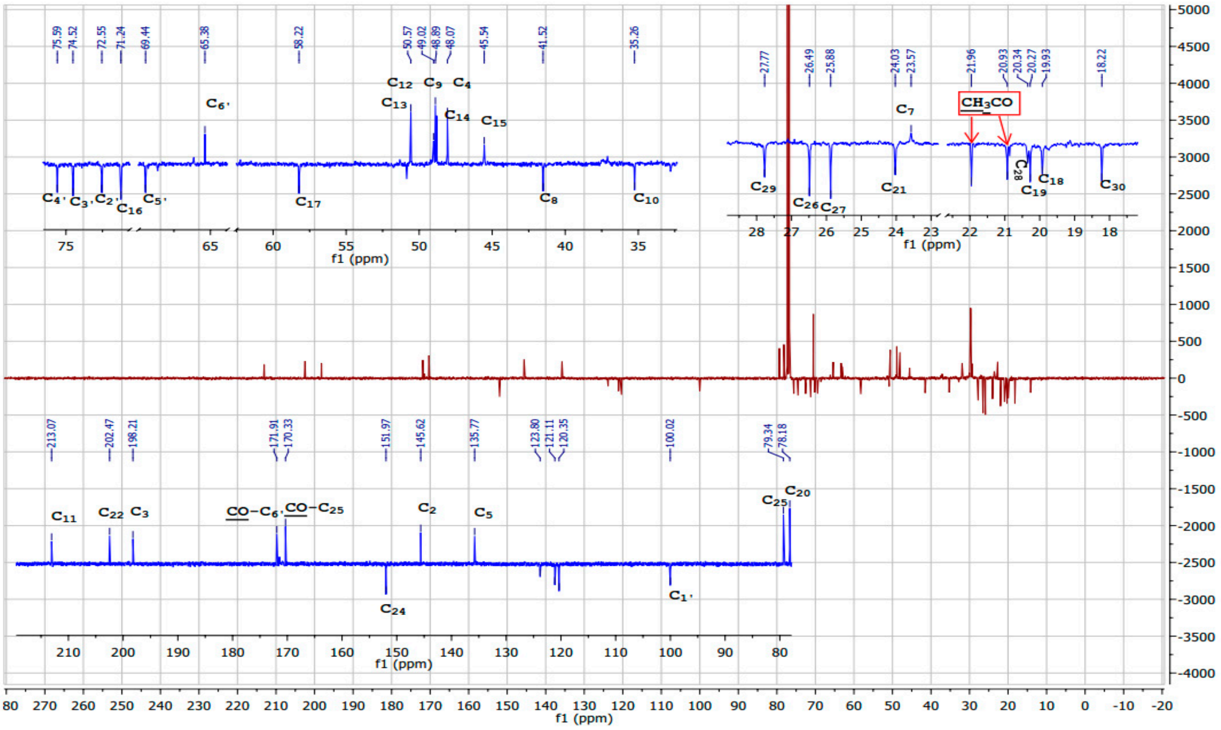

Figure S4. ^{13}C-NMR spectrum of 6′-acetyl-2-*O*-β-D-glucocucurbitacin E (**1**) at 125 MHz in $CDCl_3$.

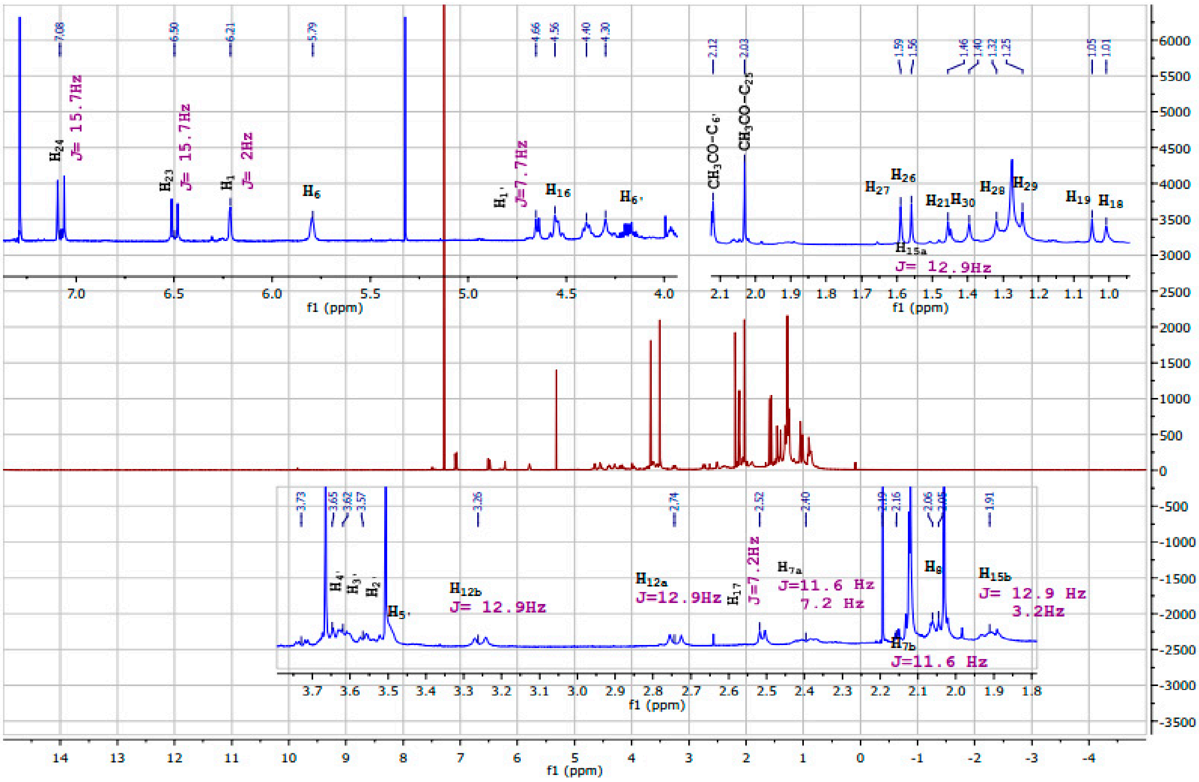

Figure S5. ^{1}H-NMR spectrum of 6′-acetyl-2-*O*-β-D-glucocucurbitacin E (**1**) at 500 MHz in $CDCl_3$.

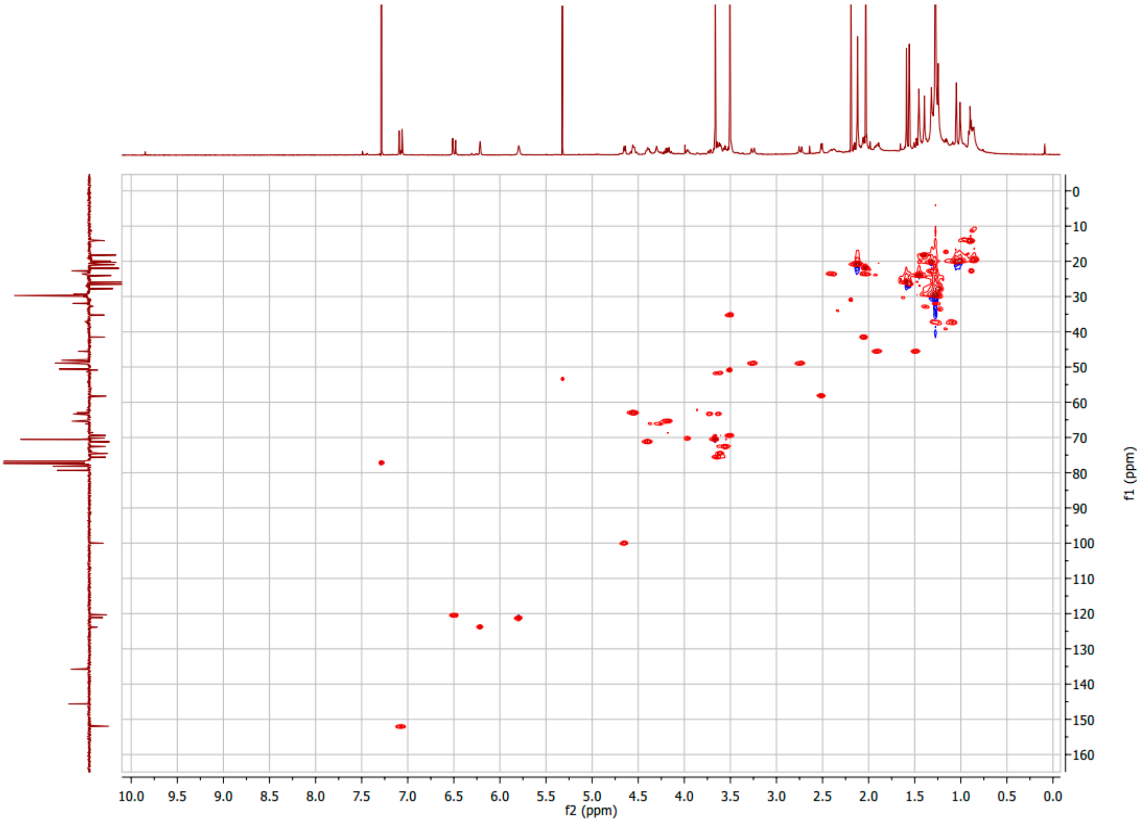

Figure S6. HMBC NMR spectrum of 6′-acetyl-2-*O*-β-D-glucocucurbitacin E (**1**) at 500/125 MHz spectrum in $CDCl_3$.

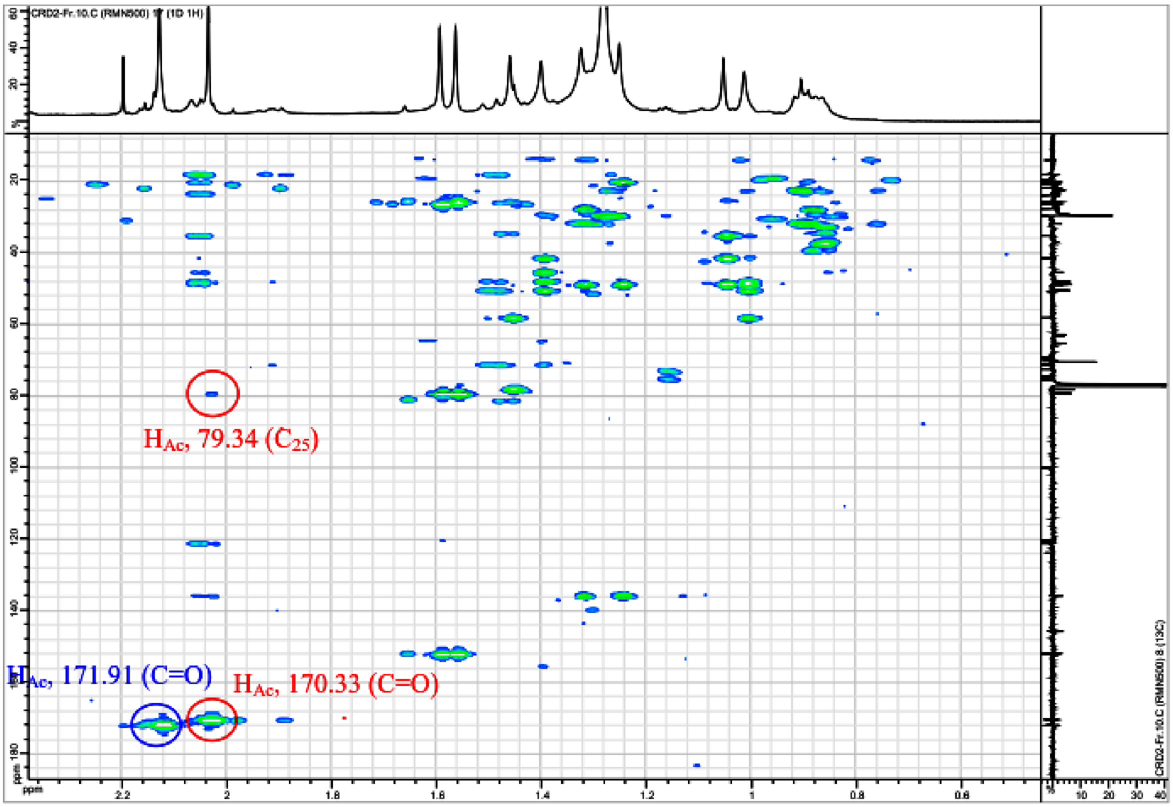

Figure S7. HMBC NMR spectrum of 6′-acetyl-2-*O*-β-D-glucocucurbitacin E (**1**) at 500/125 MHz spectrum in $CDCl_3$ (Expansion).

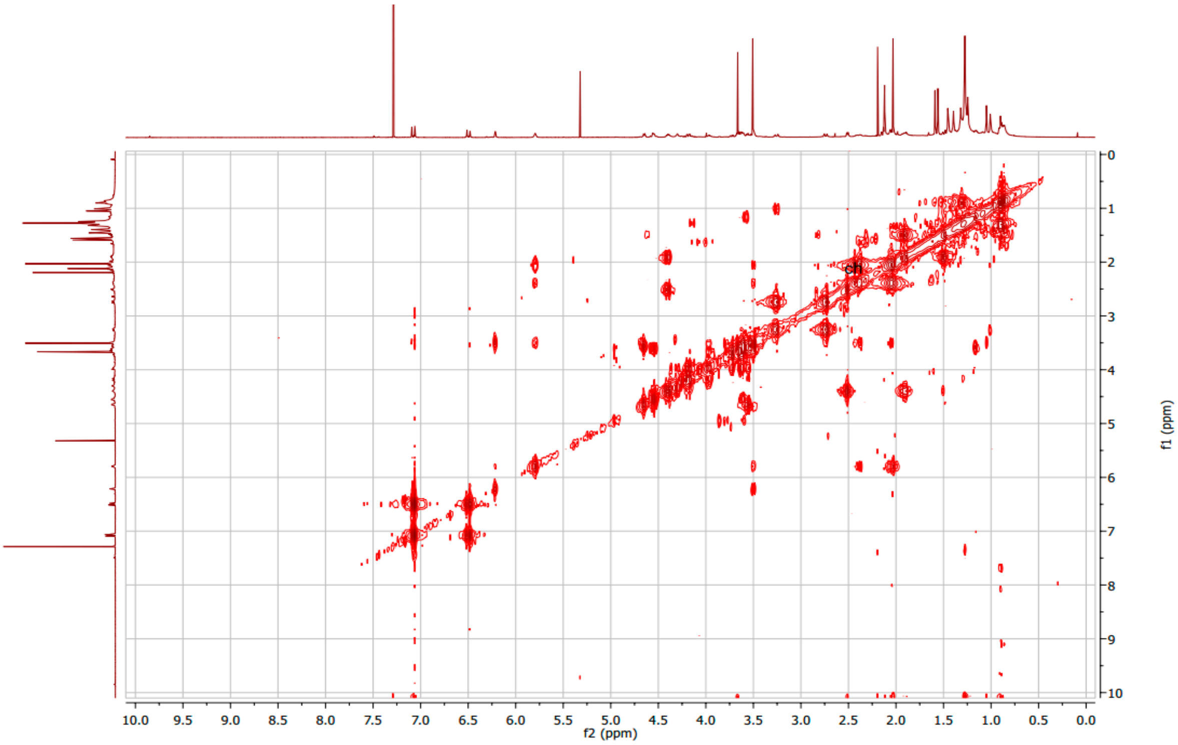

Figure S8. HSQC NMR spectrum of 6′-acetyl-2-*O*-β-D-glucocucurbitacin E (**1**) at 500/125 MHz in $CDCl_3$.

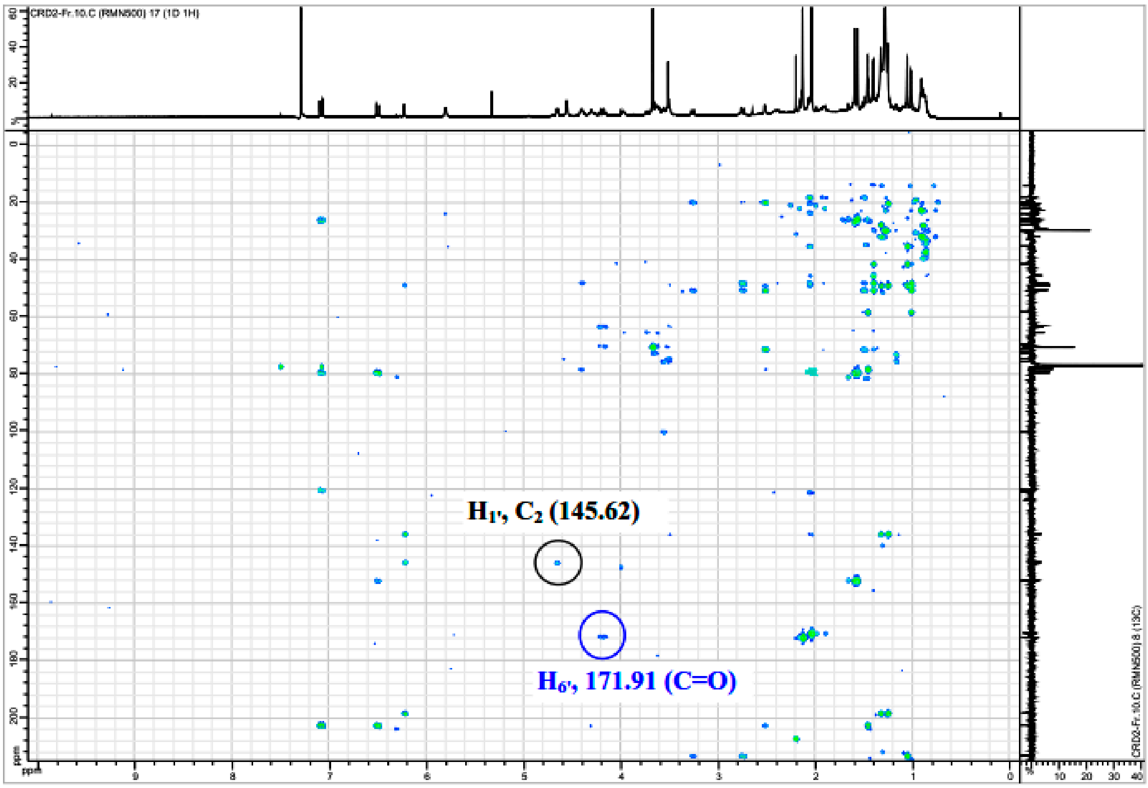

Figure S9. ^{1}H-^{1}H COSY NMR spectrum of 6′-acetyl-2-*O*-β-D-glucocucurbitacin E (**1**) at 500 MHz in $CDCl_3$.

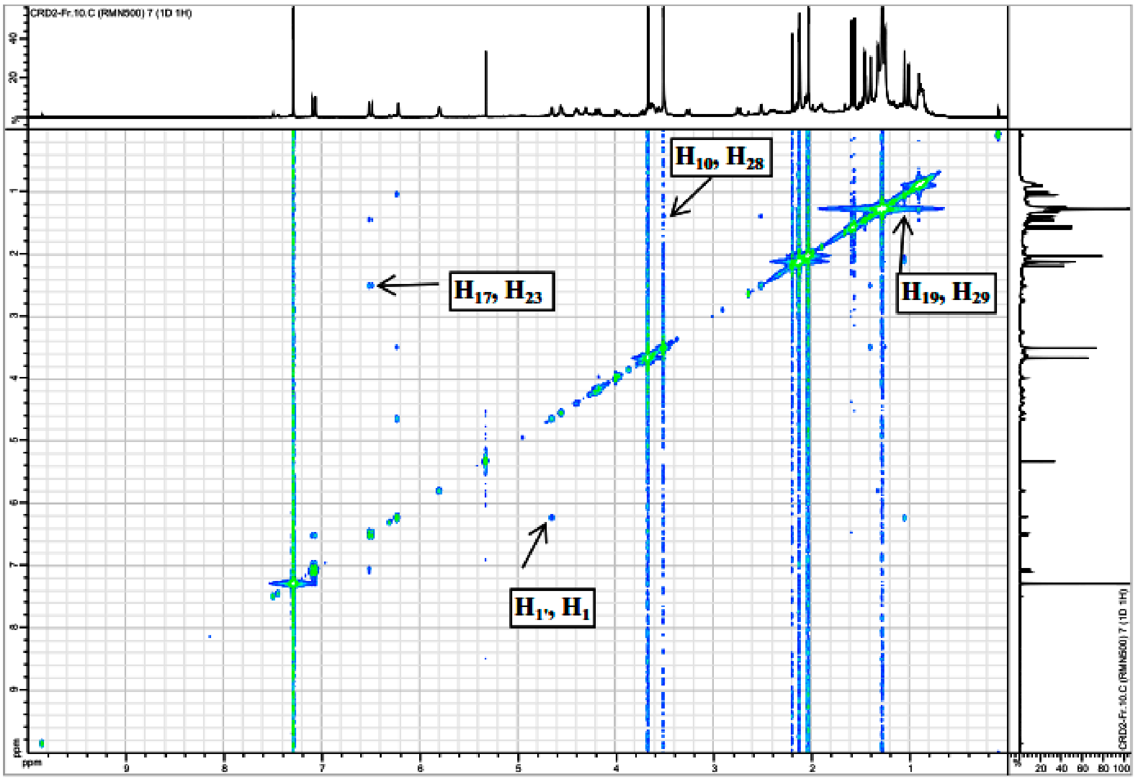

Figure S10. ^{1}H-^{1}H NOESY NMR spectrum of 6′-acetyl-2-*O*-β-D-glucocucurbitacin E (**1**) at 500 MHz in $CDCl_3$.

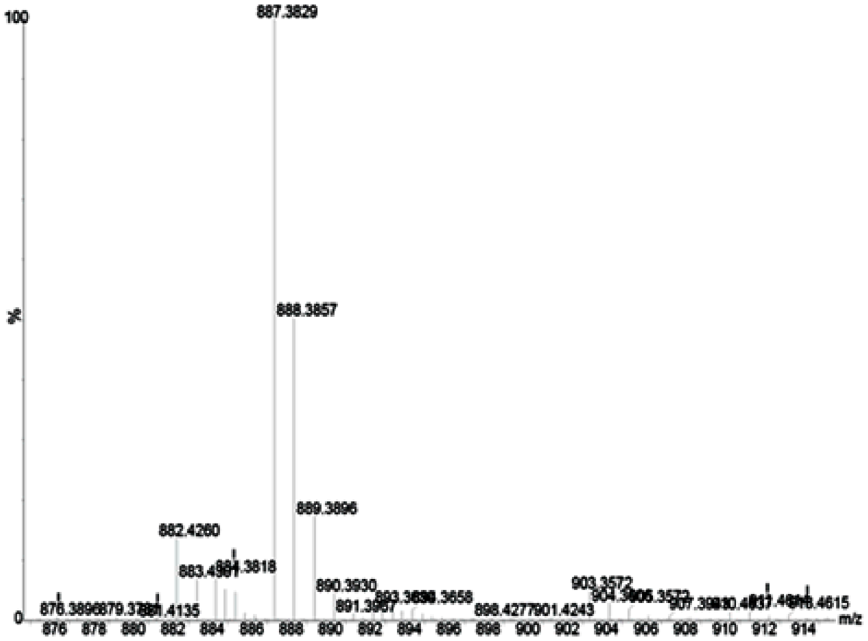

Elemental Composition Report							
Mass	Calc. Mass	mDa	PPM	DBE	i-FIT	i-FIT (Norm)	Formula
887.3829	887.3830	−0.1	−0.1	17.5	132.4	0.9	C47 H60 O15 Na

Figure S11. HR-ESI(+)MS spectrum of 25-*p*-coumaroyl-3′-acetyl-2-*O*-β-D-glucocucurbitacin I (**2**) obtained by direct infusion of a methanolic solution (100 µg/mL). Spectrum shows the *m/z* 741.3453 ion highlighting the neutral loss of the *p*-coumaroyl moiety. Elemental composition report was given by MassLynx V4.1 software.

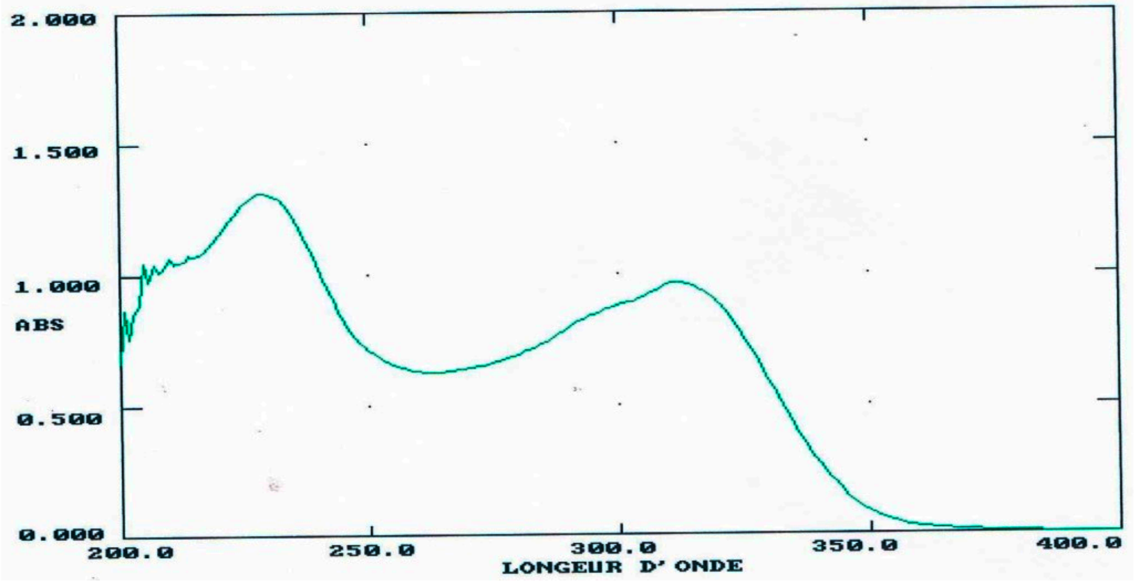

Figure S12. UV spectrum of 25-*p*-coumaroyl-3′-acetyl-2-*O*-β-D-glucocucurbitacin I (**2**) in MeOH.

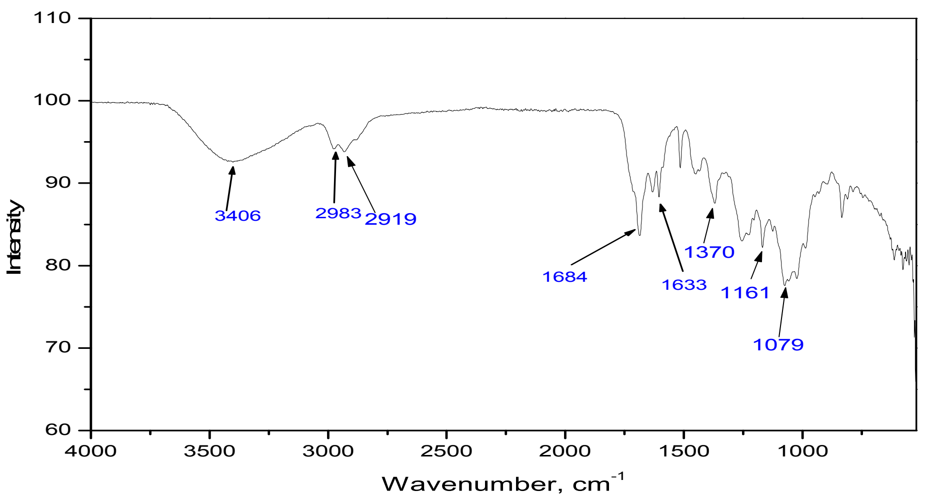

Figure S13. IR spectrum of 25-*p*-coumaroyl-3′-acetyl-2-*O*-β-D-glucocucurbitacin I (**2**) recorded on a Perkin-Elmer 100 FT-IR spectrometer.

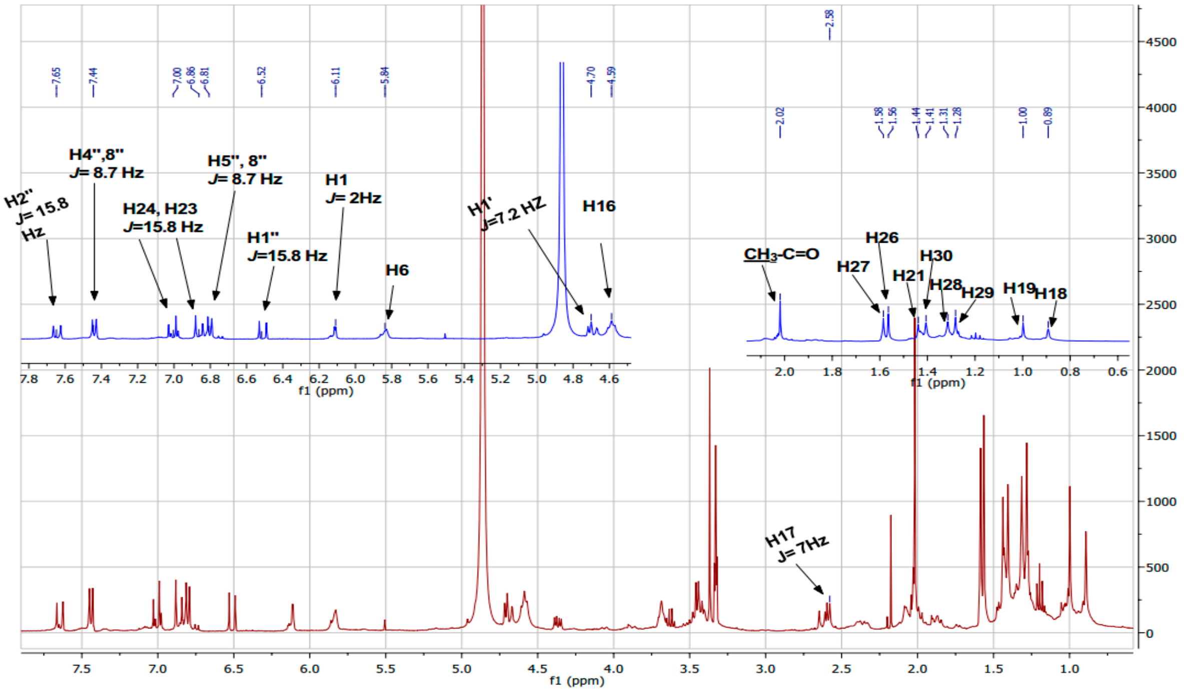

Figure S14. ^{1}H-NMR spectrum of 25-*p*-coumaroyl-3′-acetyl-2-*O*-β-D-glucocucurbitacin I (**2**) at 400 MHz in CD_3OD.

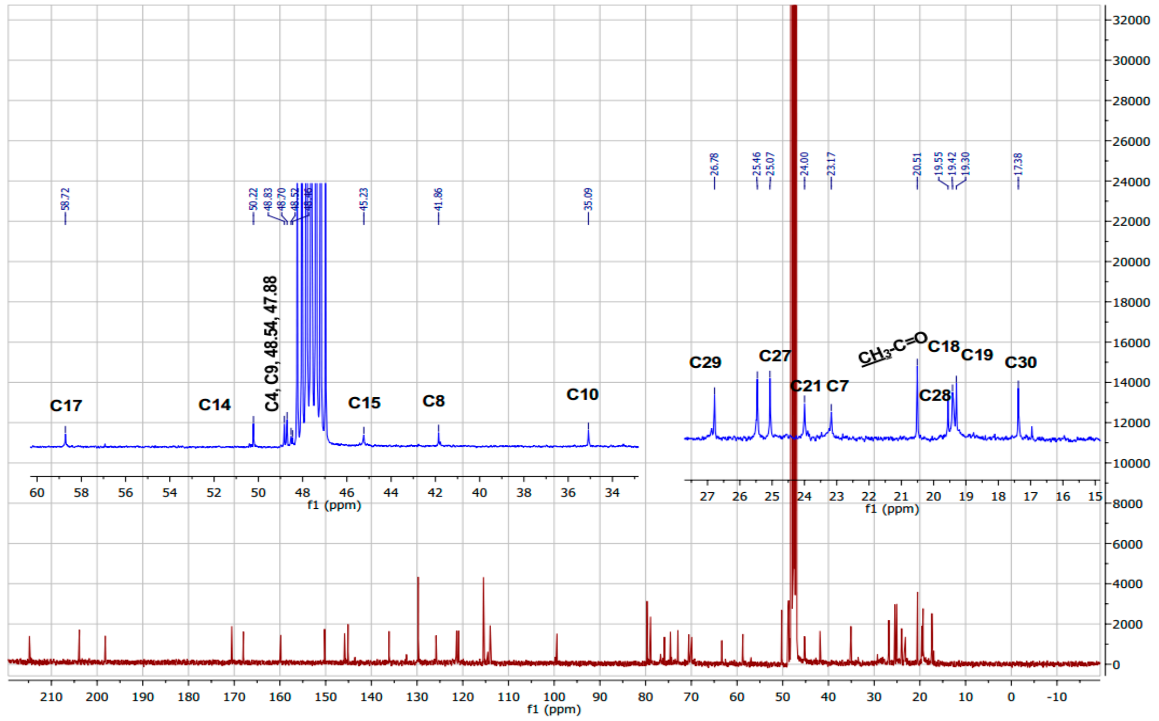

Figure S15. ^{13}C-NMR spectrum of 25-*p*-coumaroyl-3′-acetyl-2-*O*-β-D-glucocucurbitacin I (**2**) at 100 MHz in CD_3OD.

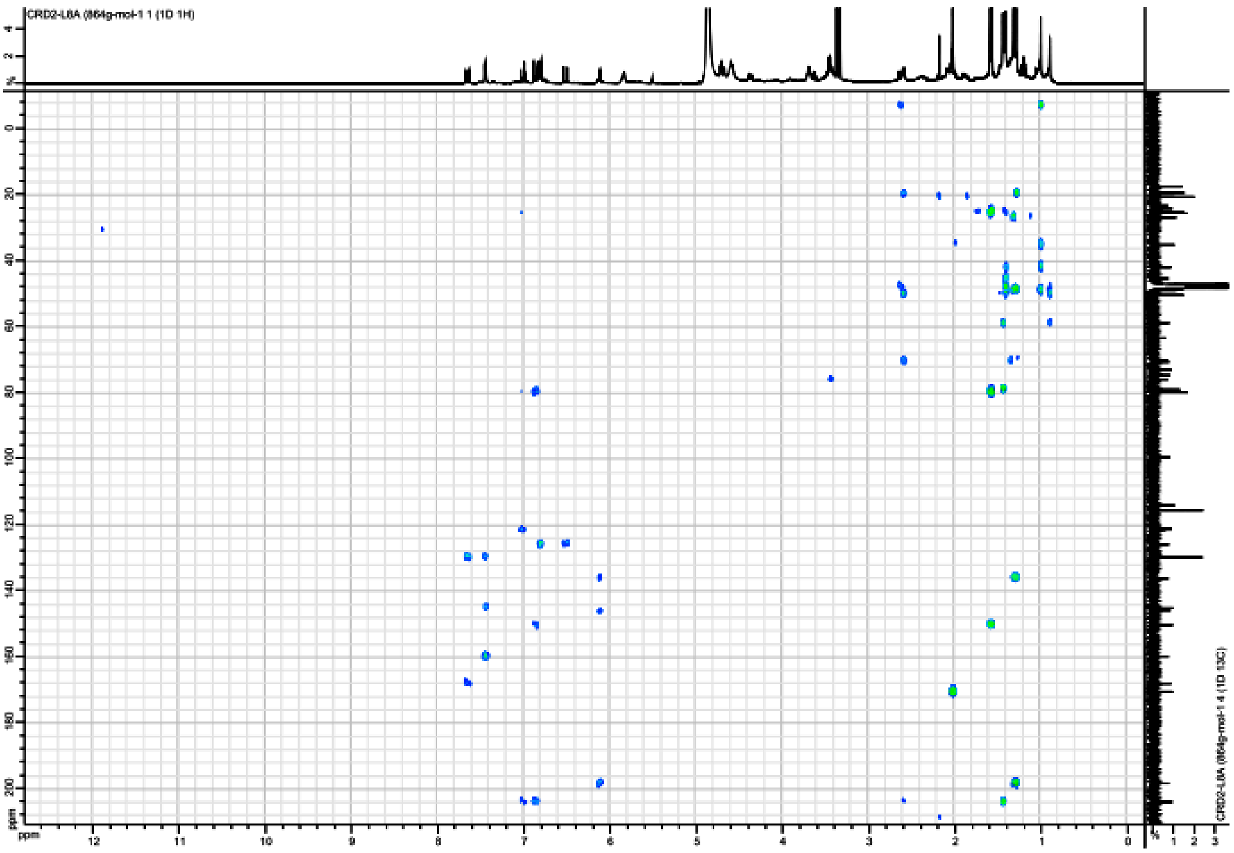

Figure S16. HMBC NMR spectrum of 25-*p*-coumaroyl-3′-acetyl-2-*O*-β-D-glucocucurbitacin I (**2**) at 400/100 MHz in CD_3OD.

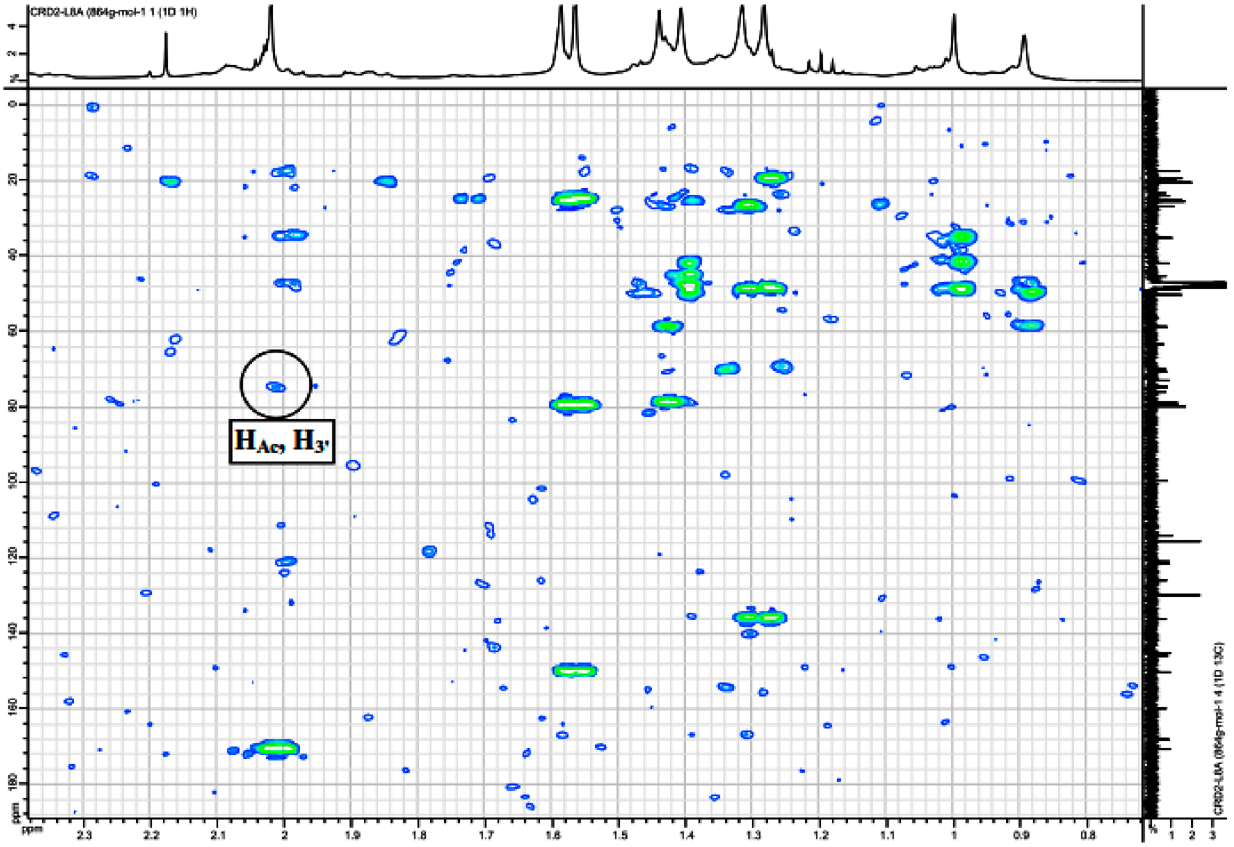

Figure S17. HMBC NMR spectrum of 25-*p*-coumaroyl-3′-acetyl-2-*O*-β-D-glucocucurbitacin I (**2**) at 400/100 MHz in CD_3OD (Expansion).

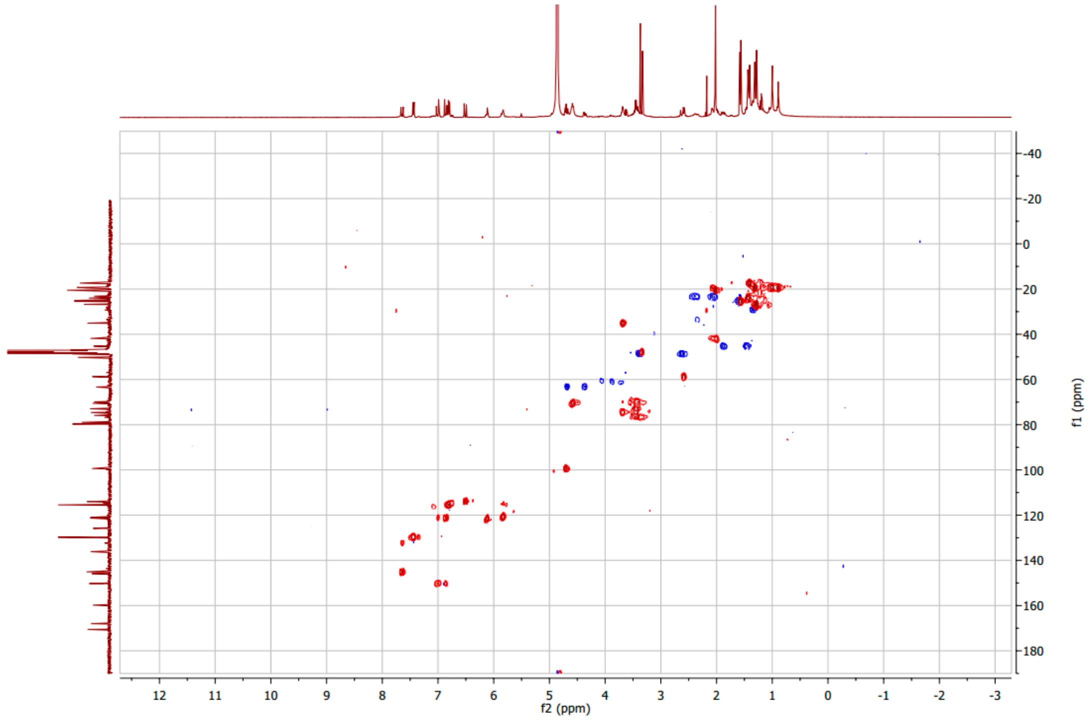

Figure S18. HSQC NMR spectrum of 25-*p*-coumaroyl-3′-acetyl-2-*O*-β-D-glucocucurbitacin I (**2**) at 400/100 MHz in CD_3OD.

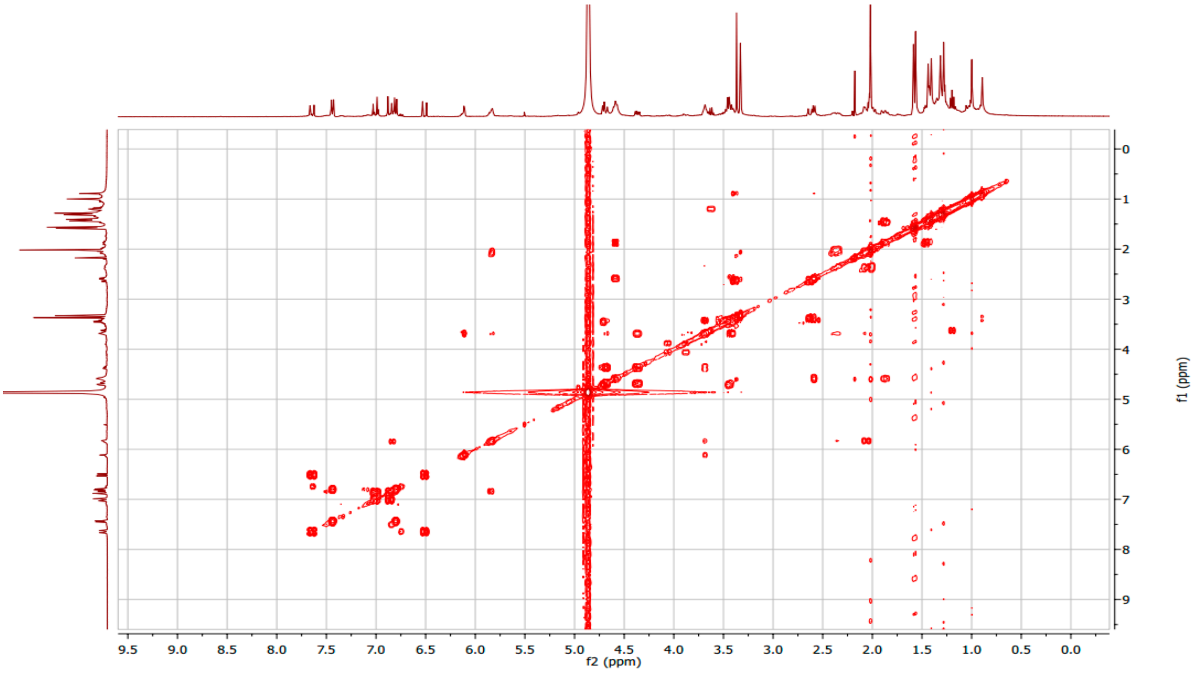

Figure S19. ^{1}H-^{1}H COSY NMR spectrum of 25-*p*-coumaroyl-3′-acetyl-2-*O*-β-D-glucocucurbitacin I (**2**) at 400 MHZ in CD_3OD.

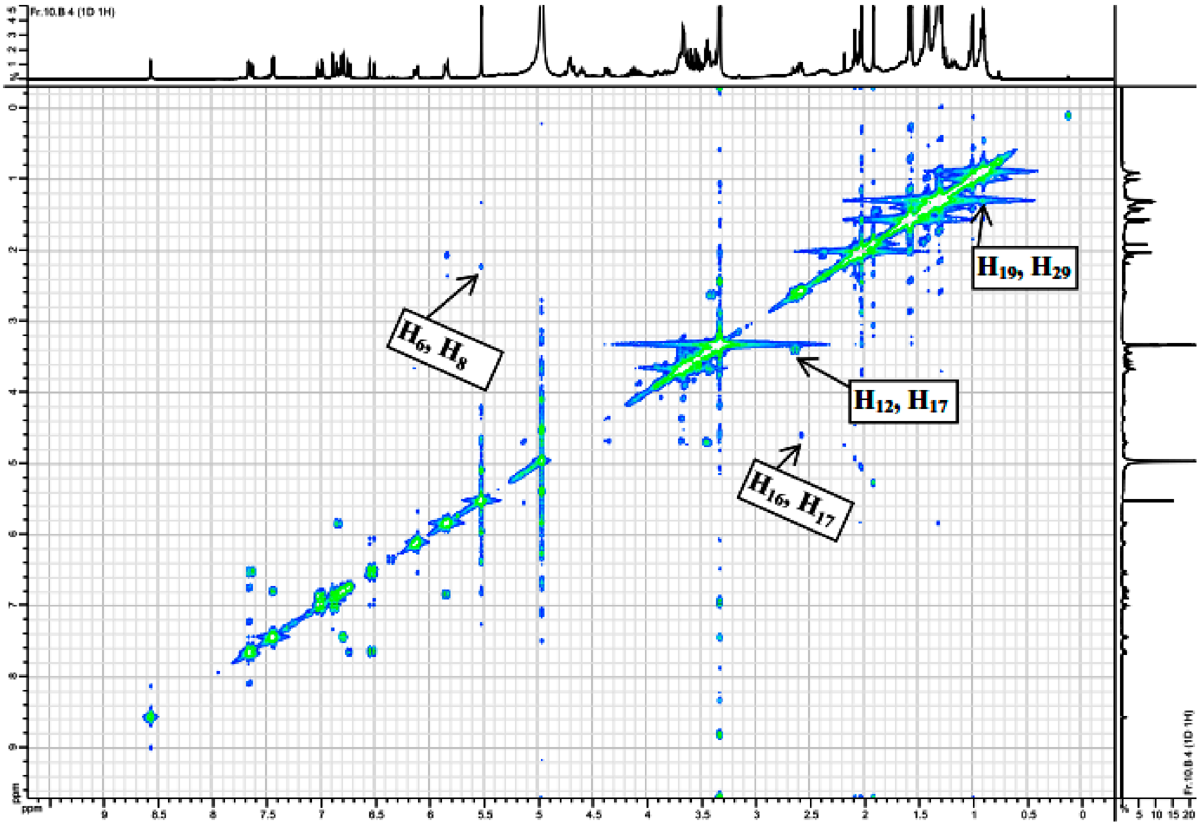

Figure S20. ^{1}H-^{1}H NOESY NMR spectrum of 25-*p*-coumaroyl-3′-acetyl-2-*O*-β-D-glucocucurbitacin I (**2**) at 400 MHz in CD_3OD.

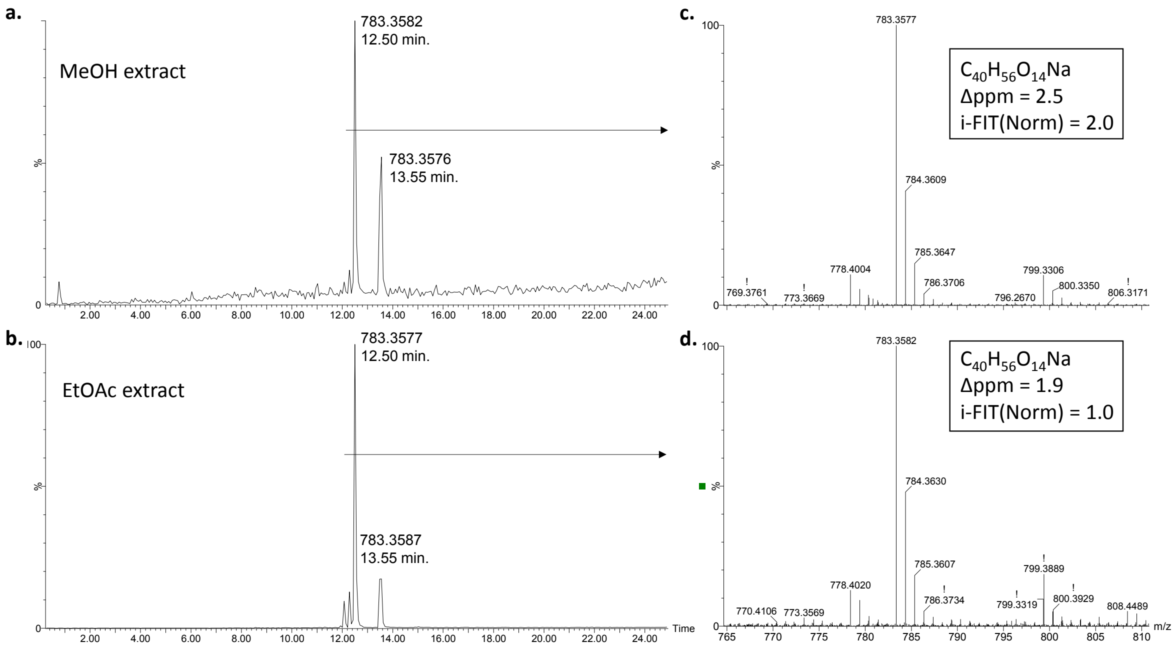

Figure S21. Extracted ion chromatograms of ion at *m/z* 783.35 (±0.02 Da) of UHPLC-TOFMS profiles from crude MeOH (**a**) and EtOAc (**b**) extracts showing two isobaric compounds at 12.50 and 13.55 min detected in both extracts. A comparison with purified compound (**1**) displayed a perfect match with retention time 12.50 min; (**c**,**d**): mass spectra of main ions detected at 12.50 min for both extracts. The insets show the results of molecular formula calculation with Δppm and isotopic pattern score.

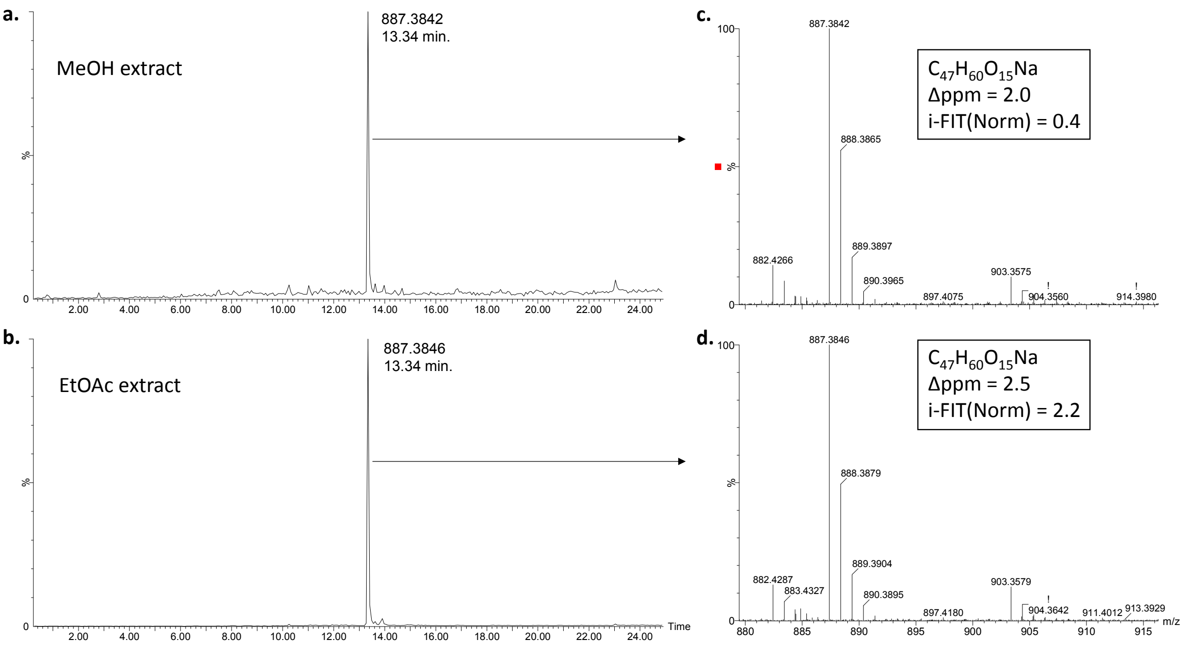

Figure S22. Extracted ion chromatogram of ion at *m/z* 887.39 (±0.02 Da) of UHPLC-TOFMS profiles from crude MeOH (**a**) and EtOAc (**b**) extracts. A comparison with purified compound (**2**) displayed a perfect match with retention time at 13.34 min; (**c**,**d**): mass spectra of main ions detected at 13.34 min for both extracts. The insets show the results of molecular formula calculation with Δppm and isotopic pattern score.
